# Significance of extracellular vesicles in orchestration of immune responses in *Mycobacterium tuberculosis* infection

**DOI:** 10.3389/fcimb.2024.1398077

**Published:** 2024-05-21

**Authors:** Shamila D. Alipoor, Daniel Elieh-Ali-Komi

**Affiliations:** ^1^ Division of Inflammation and Infection, Department of Biomedical and Clinical Sciences, Linköping University, Linköping, Sweden; ^2^ Institute of Allergology, Charité – Universitätsmedizin Berlin, Corporate Member of Freie Universität Berlin and Humboldt-Universität zu Berlin, Berlin, Germany; ^3^ Fraunhofer Institute for Translational Medicine and Pharmacology (ITMP), Immunology and Allergology, Berlin, Germany

**Keywords:** extracellular vesicles, exosomes, *Mycobacterium tubercilosis*, tuberclosis, lung cancer

## Abstract

*Mycobacterium tuberculosis (M.tb)*, the causative agent of Tuberculosis, is an intracellular bacterium well known for its ability to subvert host energy and metabolic pathways to maintain its intracellular survival. For this purpose, the bacteria utilize various mechanisms of which extracellular vehicles (EVs) related mechanisms attracted more attention. EVs are nanosized particles that are released by almost all cell types containing active biomolecules from the cell of origin and can target bioactive pathways in the recipient cells upon uptake. It is hypothesized that M.tb dictates the processes of host EV biogenesis pathways, selectively incorporating its molecules into the host EV to direct immune responses in its favor. During infection with *Mtb*, both mycobacteria and host cells release EVs. The composition of these EVs varies over time, influenced by the physiological and nutritional state of the host environment. Additionally, different EV populations contribute differently to the pathogenesis of disease at various stages of illness participating in a complex interplay between host cells and pathogens. These interactions ultimately influence immune responses and disease outcomes. However, the precise mechanisms and roles of EVs in pathogenicity and disease outcomes remain to be fully elucidated. In this review, we explored the properties and function of EVs in the context of M.tb infection within the host microenvironment and discussed their capacity as a novel therapeutic strategy to combat tuberculosis.

## 1 Introduction

Tuberculosis (TB) was the leading infectious cause of mortality globally before the COVID-19 pandemic, with an estimated 10 million infections and 1.4 million deaths in 2020 (Migliori et al., 2021). TB is caused by an intracellular bacterium called *Mycobacterium tuberculosis (M.tb).*

Our inadequate understanding of host-pathogen interactions is the main barrier to managing TB. It is well understood that *M.tb* has developed multiple intricate mechanisms to subvert the host energy and metabolic pathways ensuring its intracellular survival while evading the host immune system. These ingenious mechanisms impress several mechanisms of host immune responses to dampen inflammatory cytokines, inhibition of phagosome maturation and fusion with lysosome as well as inhibition of apoptosis or promoting the necrosis of host macrophages (Schorey and Schlesinger, 2016). In this regard *M.tb* benefits from a variety of biological systems and mechanisms of which extracellular vesicles (EVs)-related mechanisms have attracted more attention and are considered the crucial elements in the pathology of bacteria and host-pathogen interactions (Palacios et al., 2021).

EVs are produced and released by nearly all cell types and organisms and play a crucial role in cell-to-cell communication. EVs have a membrane structure generally ranging from 30 to 1000 nm in size (Gill et al., 2019). They contain different metabolites, proteins, lipids, and nucleic acids from the cell of origin and their cargo may change based on the physiologic state of the origin cell and environmental conditions. These vesicles shuttle active biomolecules, influencing a plethora of physiological functions in recipient cells (Gill et al., 2019).

During infection with intracellular pathogens including *Mycobacterium tuberculosis*, both infected host cells and the pathogens produce EVs (Gioseffi et al., 2021; Zou et al., 2022). These EVs harbor either microbial antigens or host-originated molecules influenced by the infected cell’s physiological conditions or pathological state, which strongly contribute to the immune responses (Schorey et al., 2021). It is evident that during infection, various EV populations contribute in different manners to the pathogenesis and phenotype of the disease. However, differentiation between the populations of host and *M.tb*-derived EVs is challenging with the current processing methods including sucrose gradient or ultracentrifugation methods (Mehaffy et al., 2022). Effective management of the disease will depend on our ability to comprehend the characteristics and functions of the various EV populations during tuberculosis infection.

In this review, we examine the characteristics and roles of EVs concerning *M.tb* infection within the host environment and explore how they can be used as a new way to treat tuberculosis.

## 2 A brief overview of the properties of the EVs during the infection with *mycobacterium tuberculosis*

### 2.1 Bacterial-derived EVs

#### 2.1.1 Biological properties and content

EVs produced by pathogenic bacteria influence host immune responses via shuttling the virulence factors including enzymes (Galka et al., 2008; Bomberger et al., 2009), toxins (Kato et al., 2002; Wai et al., 2003), and lipopolysaccharide (LPS) (Kato et al., 2002) in concentrated amounts and protect them against solubility or dilution through diffusion (Deatherage and Cookson, 2012).

Production of bacterial vesicles was first described about 60 years ago in Gram-negative bacteria, *Escherichia coli (E.coli)* (Work et al., 1966). However, in the case of mycobacteria, the report of their production dates back to 2007 when their role in biofilm formation was reported in the *M. ulcerans* (Marsollier et al., 2007). In this study, scanning electron microscopy (SEM) imaging showed the presence of vesicles associated with the biofilm. This led to the first purification and immunoprecipitation of mycobacterial EVs from infected mice tails. These EVs contained the lipidic toxin mycolactone which is one of the important virulence factors of *M. ulcerans* (Marsollier et al., 2007). Mycolactone specifically targets the membrane channel Sec61, which interferes with the production of transmembrane and secreted proteins including cytokines and immune-related components (Nitenberg et al., 2018; Foulon et al., 2020).

Subsequently, the production and release of EVs were detected in other mycobacterial species including pathogenic *Mycobacterium avium* subspecies *paratuberculosis* (MAP) and *Mycobacterium avium* subspecies *hominissuis* (MAH). It has been shown that the production of EVs is conserved in almost all species of mycobacterium, however, the kinetic of EVs production differs between species (Prados-Rosales et al., 2011).

In the case of *M.tb*, the membrane of the released EVs included polar lipids from the bacterial inner cytoplasmic membrane including phosphatidylinositol (PI), phosphatidylinositol mannosides (PIMs), phosphatidylethanolamine (PE), and cardiolipin, accompanied by the lipoglycan Lipoarabinomannan (LAM). This indicated the inner cytoplasmic membrane as the origin of *M.tb*-released EVs (Prados-Rosales et al., 2011).

Extensive proteomic and lipidomic analysis of the components from these EVs demonstrated enrichment of phospholipids and lipoprotein as well as the high presence of a variety of toll-like receptor 2 (TLR-2) ligands in these EVs including LpqH, Heat shock proteins(HSPs), LprG, LprA, and LAM (Prados-Rosales et al., 2011). Additionally, bacterial genomic double-strand DNA (dsDNA) has been reported to be present inside the bacterial EVs (Chiplunkar et al., 2019). While the presence of mycobacterial transcripts has been detected in the EVs derived from the *M.tb*-infected macrophages, very little RNA was detected in the bacterial EVs obtained from the *M.tb* culture (Singh et al., 2015).

#### 2.1.2 EVs as a survival mechanism of M.tb in harsh conditions

Although the presence of a cluster of special molecules inside bacterial EVs has been reported and confirmed, the composition of these EVs and their biological properties may vary across different host microenvironments such as in the food or energy deprivation condition (Chiplunkar et al., 2019). It has been demonstrated that bacterial EVs play an important role in managing harsh conditions by bacteria.

In a study by Chiplunkar, the formation and composition of EVs from *Mycobacterium avium* were investigated in a model that mimicked the environment of macrophage phagosome. Exposure of the bacteria to metal concentrations found in phagosomes triggered the formation of bacterial EVs with potential roles in the modulation of host immune responses and bacterial intracellular survival. Proteomics identified the presence of several virulent factors inside these EVs including enzymes responsible for the metabolism of lipids, fatty acids, and cell wall synthesis. The protein composition of these vesicles varied in different nutrition conditions, which provides additional evidence that mycobacterial EVs play a role in responding to changes in nutrient availability within the environment (Chiplunkar et al., 2019).

On the other hand, the EVs released by Mycobacterium have a role in iron acquisition for bacteria (Prados-Rosales et al., 2014). Iron plays a crucial role in the outcome of mycobacterium infections. However, the bacteria face the challenge of iron limitation within host-infected cells due to iron’s poor solubility at the host’s biological pH. In fact, in mammalian host cells, Iron remains bound to specialized iron-binding proteins such as transferrin, lactoferrin, and ferritin (Rodriguez et al., 2022). To overcome this limitation, the pathogen produces siderophores, specifically mycobactin, to chelate iron ions. Mycobactin exhibits an exceptionally high affinity for Fe^3+^ and can extract it from insoluble components and iron-binding proteins (Gobin and Horwitz, 1996; Sritharan, 2016). Mycobactin possesses a long alkyl chain substitution, rendering it highly hydrophobic (Messenger and Ratledge, 1982) which potentiates its packaging into EVs for secretion (Prados-Rosales et al., 2014).

Under iron limitation mycobacteria increase production and release EVs loaded with mycobactin (Prados-Rosales et al., 2014). Iron deprivation in *M.tb* cells induces iniBAC operon activation which in turn encodes the large GTPases dynamin-like proteins (DLPs) IniA and IniC, to increase the release of EVs by *M.tb* (Gupta et al., 2020). These EVs can support the growth of iron-deprived bacteria and rescue the *M.tb* mutant with a deficiency in making mycobactin (Prados-Rosales et al., 2014).

The process of biogenesis of mycobacterial EVs is also regulated by vesiculogenesis and immune response regulator (virR) (Rath et al., 2013), and the Two-component system SenX3-RegX3 (White et al., 2018). While iron limitation and signaling of the SenX3-RegX3 increase the release of EVs, virR expression reduces vesicle production (Rath et al., 2013).

Interestingly it was also shown that isoniazid (INH, the first-line antibiotic used in tuberculosis treatment) can induce expression of iniBAC operon in the subinhibitory concentrations (Gupta et al., 2020). Based on these findings, a suboptimal exposure of *M.tb* to INH in the host cells might increase the effects of Mycobacterial EVs on the immune system by enhancing vesicle production (Gupta et al., 2020).

Increasing the release of EVs in mycobacteria in the presence of antibiotics may be a strategy used by bacteria to compensate for the antibiotic effects through suppression of the immune responses and enhancing replication. Therefore, it appears that control over the production, contents, and release of EVs in harsh conditions or different host microenvironments is a tactic used by bacteria for the maintenance of their intracellular survival.

### 2.2 Host-derived EVs

As an airborne pathogen, *M.tb*, besides exposure to airway epithelial cells, exposes to and infects alveolar macrophages where *M.tb* establishes an ideal niche for replication (Singh et al., 2012). Bacterial proliferation induces the production of a pro-inflammatory response, and subsequently recruitment of additional macrophages and immune cells, which results in the formation of a granuloma. With developing host adaptive immunity, the granuloma can limit the growth and expansion of bacteria. Nevertheless, within the granuloma, certain cells may undergo necrosis and create a necrotic core where *M.tb* can persist extracellularly (Ramakrishnan, 2012). On the other hand, during the infection, the pathogen also may reside in the phagolysosome which is formed by the fusion of the lysosome and the phagocytic vacuoles (Pethe et al., 2004). Shortly after infection, the mycobacterial components including cell wall components, lipoproteins, or glycolipids exit the phagosome through endocytic compartments and are subsequently released within host-derived EVs (Palacios et al., 2021). These EVs mostly share similar size and surface markers with exosomes (Prados-Rosales et al., 2011; Smith et al., 2015) and their components and properties have been studied in various experimental models including infected cells, animal models as well as in serum or plasma of tuberculosis patients (Giri et al., 2010; Kruh-Garcia et al., 2014; Singh et al., 2015; Mehaffy et al., 2017). Even though different studies have found different kinds and amounts of components inside these EVs, there is a consensus for the enrichment of the TLR2 ligands in the infected host-derived EVs, like those in bacterial-derived EVs. Many of these components have been characterized as highly immunogenic mycobacterial antigens including antigen 85 (Ag85) complex, early secreted antigenic target of 6 kDa (ESAT-6), and MPT63 (Rath et al., 2013).

Although there are some overlaps between bacterial components inside the EVs from the infected host or bacteria, interestingly, most of the mycobacterial proteins found in host-derived EVs are not present in bacterial-derived EVs (Kruh-Garcia et al., 2014; Mehaffy et al., 2022). So, it is worth knowing how bacterial components are targeted to host EVs in the infected cells. To address this question, Smith et al. looked at the role of the ubiquitylation process in this context. Interestingly, when mycobacterial proteins were introduced separately to both RAW264.7 mouse macrophages and Human Embryonic Kidney (HEK) 293 cells, they underwent endocytosis, ubiquitination, and subsequent release via host EVs. While suppression of the ubiquitylation using its corresponding inhibitors led to the loss of mycobacterial components in host-derived EVs. Furthermore, ubiquitylated proteins were found to bind to the endosomal sorting complexes required for transport (ESCRT-I) for their dissemination into MVBs (Singh et al., 2023). These findings suggested that ubiquitination is a necessary process for trafficking bacterial components from the phagocytic/endocytic network to host EVs (Smith et al., 2015). Interestingly *M.tb* can manipulate these processes by inducing the expression of the host ESCRT-dependent and independent genes in the infected cells and increasing the production of EVs with different protein and lipid composition in different stages of disease (Singh et al., 2023).

Characterization and separation of host and *M.tb*-derived vesicles based on their content and function can be informative in this regard. Athman et al. tried to separate two populations of bacterial EVs and host-derived EVs within macrophage culture supernatants using a sucrose density gradient based on their specific surface markers (Athman et al., 2015). However, their conclusion was challenging, and based on the data provided it was not possible to confirm that human and *M.tb* EVs were completely separated in their experiments. So further experiments using progressive techniques including advanced flow cytometry methods may help for efficient separating of different populations of EVs.

## 3 The mechanisms of EVs in modulation of immune responses during infection with *M.tb*

EVs released by the bacteria or infected host cells can have either inflammatory or modulatory effects on immune response against *M.tb* during the infection. EVs, manipulate immune response during *M.tb* infection by interfering with different immune mechanisms including secretion of chemokines, expression of costimulatory surface receptors, function, activity, and recruitment of immune cells including neutrophils, macrophages, and splenocytes (Singh et al., 2011; Singh et al., 2012; Ziegenbalg et al., 2013; Wang J. et al., 2015; Nitenberg et al., 2018).

### 3.1 Modulation of inflammatory responses and the role of TLRs

The importance of EVs released by Mycobacterium-infected cells in immune response regulation during intracellular bacterial infections was first reported by Sanchita Bhatnagar and colleagues (Bhatnagar and Schorey, 2007). They demonstrated that *M. avium*-infected macrophages released EVs containing glycopeptidolipids (GPLs) and could transfer these molecules from infected to uninfected macrophages and trigger proinflammatory responses by these macrophages (Bhatnagar and Schorey, 2007). Subsequently, they identified some of the antigenic mycobacterial components, including LAM and the 19-kDa lipoprotein, within the EVs purified from the bronchoalveolar lavage fluid (BALF) of BCG-infected mice (Bhatnagar et al., 2007). These EVs had proinflammatory properties and when applied intranasally into mice, they stimulated TNF and IL-12 production and enhanced the migration of neutrophils and macrophages to the lung of the animal (Bhatnagar et al., 2007). Interestingly, the proinflammatory properties of these EVs were dependent on the time of isolation. EVs isolated within 48- and 72 hours post-infection showed stimulatory activity, while at earlier or later time points, the isolated EVs failed to trigger the inflammatory responses. In addition, the stimulatory activity correlated with the levels of exosomal LAM and 19-kDa lipoprotein which suggested that the trafficking of mycobacterial components to multivesicular bodies (MVB) changed over time (Bhatnagar et al., 2007).

These studies for the first time highlighted the important role of EVs as the carrier of Pathogen-Associated Molecular Patterns (PAMPs) in the mechanism of immune responses against intracellular pathogens.

**Figure 1:**
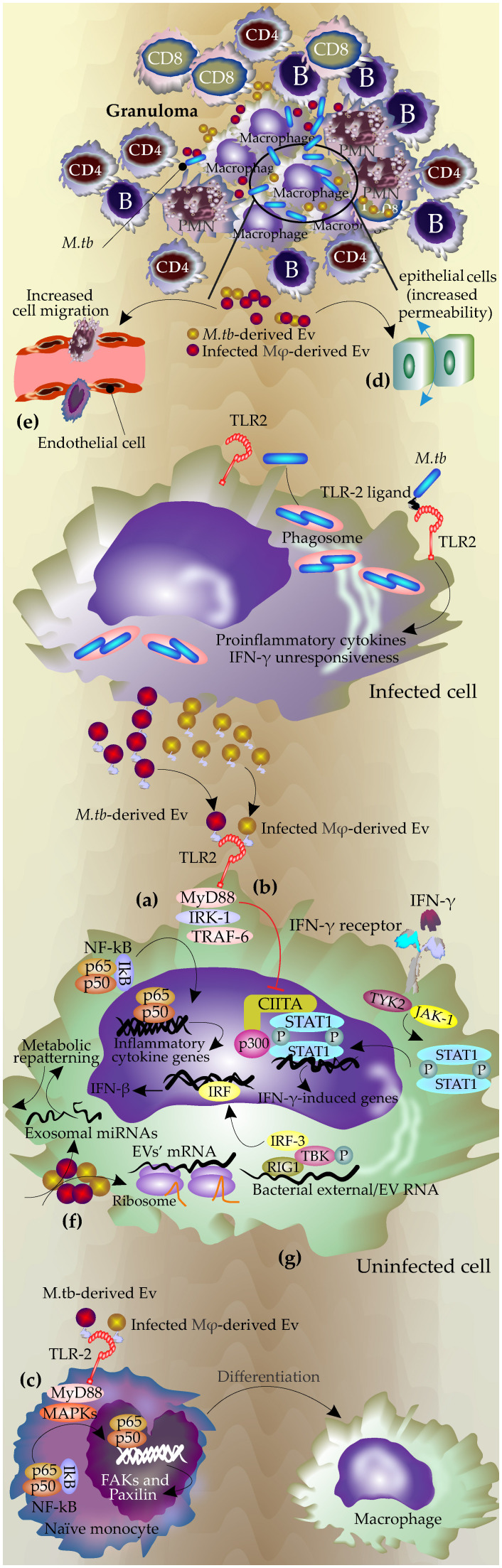
The mechanisms of EVs during infection with Mycobacterium tuberculosis. During *M.tb* infection, the pathogen resides in the phagolysosome, and the mycobacterial components, including antigen molecules, exit the phagosome either via host-derived EVs or bacterial ones. Bacterial-released EVs can be produced inside the phagosomes or by extracellular mycobacteria in granuloma. **(A)** Bacterial and host-derived EVs contain TLR ligands and activate TLR signaling pathways in uninfected cells, leading to the production of proinflammatory cytokines by the uninfected cells through NF-κB pathways. **(B)** These EVs block the response to interferon-gamma in the uninfected cell by inhibiting the corresponding genes at the transcriptional level by downregulation of transcription factor CIITA and its interaction with p300 (Ting et al., 1999; Singh et al., 2011) **(C)** Promoting macrophages differentiation through TLR and MPK signaling pathways, which lead to increasing FAX and paxillin expression and actin polymerization. **(D)** Increasing permeability and cell migration in the epithelial cells. **(E)** Activation of endothelial cells by increasing MMPs expression and permeability of the monolayer and finally increasing the cell migration. **(F)** mRNAs from these EVs are active and can be translated in recipient cells. **(G)** RNAs from these EVs may activate RNA-sensing machinery in recipient cells.

Furthermore, when macrophages from TLRs or MyD88-knockout mice were incubated with these EVs, there was no induction of the proinflammatory response which demonstrated that the mechanism underlying the proinflammatory activity of these EVs is dependent on TLRs and MyD88-related pathways and also highlighted that these EVs contained TLR ligands (Bhatnagar and Schorey, 2007; Bhatnagar et al., 2007) (**Figure 1A**). Interestingly, proteomic analysis showed that only EVs from the virulent strains contained TLR2 ligands (Prados-Rosales et al., 2011).

The determinant role of EVs’ TLRs in the modulation of immune response in the recipient cells was further confirmed by other studies. Singh, P.P., et al. showed these EVs can modulate IFN-γ related pathways in the bystander cells via modulation of TLR2 and Myd88-related pathways (Singh et al., 2011). Macrophages infected with *M.tb* gain resistance to IFN-γ stimulation (Ting et al., 1999). However, this effect is not limited to infected macrophages and can gain access to uninfected macrophages via EVs (Singh et al., 2011).

IFN-γ regulates host anti-TB responses by different mechanisms including the production of reactive nitrogen and oxygen species, upregulating antigen-presenting molecules including MHC molecules as well as induction of autophagy in the infected macrophages (Ghanavi et al., 2021). Interestingly, this study revealed that the EVs released by *M.tb* H37Rv-infected macrophages inhibited these mechanisms in the uninfected macrophages by suppressing their corresponding responsible genes at the transcriptional level and acting via TLR2 and Myd88-related pathways (Singh et al., 2011)(**Figure 1B**).

Accordingly, Liu et al. showed that EVs secreted by *M.tb*-infected mesenchymal stem cells (MSCs) also triggered TLR2-Myd88 related pathways in the naïve macrophages which consequently enhanced the level of proinflammatory cytokines such as TNF, RANTES(CCL5), and iNOS (Liu et al., 2021).

EVs secreted by the *M.tb* infected neutrophils applied the same mechanism to activate naïve macrophages. These EVs triggered an inflammatory response in macrophages via TLR2/6 ligands, leading to increased expression of CD80 and PD-L1 on immature monocyte-derived dendritic cells (DCs). Additionally, they enhanced the permeability of epithelial monolayers and induced immune cell migration to the injection site in mice. Furthermore, these exosomes promoted macrophage autophagy and superoxide anion production, ultimately reducing intracellular mycobacterial survival (González-Cano et al., 2010; Walters et al., 2013; Alvarez-Jiménez et al., 2018).

### 3.2 Modulation of macrophage polarization

Studies by Singh et al. showed that exosomes released from H37rv (*M.tb*)-infected macrophages after stimulation of TLRs, triggered MAPK-dependent signaling mechanisms through MK-2 and NF-κβ activation. This process then effectively induced the differentiation of naïve monocytes to the functionally active macrophages (Singh et al., 2023)(**Figure 1C**). MyD88-dependent activation of MAPK via TLR2 signaling in addition to upregulation of the inflammatory cytokines led to actin cytoskeleton reorganization and the assembly/disassembly of focal adhesions (FAs), cell attachment, and ultimately generation of mature functional macrophages (Singh et al., 2023). The effects of inflammatory exosomes on the polarization of macrophages were also reported by other studies. The exosomes secreted by *Brucella melitensis*-infected macrophages increased the polarization of macrophages towards M1 subtype. These exosomes contained Brucella antigen components and stimulated secretion of M1 cytokines (TNF and IFN-γ) through the NF-κB signaling pathway, inhibited the secretion of M2 cytokines (IL-10), and subsequently reduced the intracellular survival of Brucella (Wang et al., 2023).

### 3.3 Immune cell migration

EVs derived from the *M.tb*- infected macrophages, disrupted the integrity of the monolayer of epithelial cells which subsequently increased the migration of inflammatory cells to the infection site in lung (Distler et al., 2006)(**Figure 1D**). Furthermore, it has been shown that these EVs can activate endothelial cells (Li et al., 2018). Endothelial cells have an important role in regulating immune responses through the regulation of leukocyte adhesion, cell migration as well as secretion of some inflammatory cytokines such as TNF (Shao et al., 2020; Mostmans et al., 2021). Treatment of endothelial cells with *M.tb*-infected macrophage-derived EVs or serum of *M.tb*-infected mice increased cell monolayer permeability. This treatment changed the expression of the genes involved in cell adhesion and inflammatory-related pathways in the endothelial cells and subsequently increased macrophage migration through the endothelial cell monolayers (Li et al., 2018)(**Figure 1E**). The process may be mediated by NF-κB since incubation with *M.Tb*-EVs, induced NF-κB nuclear localization in the endothelial cells (Li et al., 2018).

### 3.4 Stimulating the host RNA sensing pathways

EVs produced by *M.Tb*-infected macrophages contain active mRNA and miRNA which were found to be specific to these infected cells including transcripts involved in the immune responses. Such exosomal RNAs were biologically active and could be expressed in recipient macrophages (**Figure 1F**). These active RNA molecules had mostly host origin but also included mycobacterial transcripts (Singh et al., 2015).

It was shown that the host EVs containing mycobacterial RNA stimulated the cytoplasmic RNA sensor RIG-I in the recipient cells and promoted the maturation of phagosomes and bacterial clearance (Cheng and Schorey, 2018; Cheng and Schorey, 2019). It was shown that *M.tb* releases its RNA into the host-derived EVs using the mycobacterial SecA2 and ESX-1 secretion systems which subsequently activates host Mitochondrial antiviral-signaling protein (MAVS)-dependent RNA sensing machinery which consequently induces the production of IFN-β in the recipient macrophages (Cheng and Schorey, 2018; Cheng and Schorey, 2019). TANK-binding kinase 1(TBK1) and Interferon regulatory factor 3 (IRF3) are two important molecules downstream of the RNA sensing machinery that are phosphorylated and transported into the nucleus upon stimulation by external RNA(e.g. viral RNA) to initiate transcription of type I IFNs (Li et al., 2018). (**Figure 1G**).

The EVs from the infected macrophages induced phosphorylation of TBK1 at Ser172 and subsequently IRF3 nuclear translocation (Pilli et al., 2012). This process also may play a role in the activation of autophagy in host cells as the production of type I interferon in the recipient macrophages, promotes the LC3-associated phagocytosis pathway and increases phagosome maturation and *M.tb* clearance (Martinez et al., 2015). Different mechanisms of function of EVs during *M.tb* infection are summarized in **Figure 1**.

### 3.5 Modulation of T cell activation and function

During infection with *M.tb*, DCs transport bacteria to local lymph nodes. However, infected macrophages poorly present antigens to CD4+ T cells to activate them. Therefore, there should be some mechanisms taken by bacteria for manipulation of the antigen presentation process in the infected host cells (Elieh Ali Komi et al., 2018).

Walters et al. observed that microparticles (MPs) released from BCG and *M.tb*-infected macrophages activated *M. tb*-specific CD4(+) T cells. These MPs contained mycobacterial proteins and stimulated Ag-specific CD4+ T cell proliferation *in vitro* and *in vivo* in a DC-dependent manner (Walters et al., 2013).

Vázquez-Flores et al. reported the presence of mycobacterial proteins containing epitopes for CD4+ T-cell activation with high concentration in the EVs released by the *M.tb*-infected human neutrophil (Vázquez-Flores et al., 2023).

However, Athman et al. observed that direct exposure to *M.tb*-released EVs inhibits T cell activation which was demonstrated with downregulation of IL-2 production and T cell proliferation (Athman et al., 2017). *M.tb* derived EVs also were shown to induce partial anergy demonstrated by increased expression of gene related to anergy in lymphocytes (GRAIL) during primary stimulation of naïve T cells which subsequently transiently inhibited effector T cells (Athman et al., 2017).

Interestingly, in a study by Srivastava et al., it was demonstrated that *M.tb* antigens are exported from infected DCs which will be uptaken by kinesin 2-dependent vesicular transport. This process reduces the quantity of the antigens available for presentation by MHC class II-related and subsequently, limits CD4 T cell activation (Srivastava et al., 2016). locking the release of “free” antigens by silencing kinesin-2 in the *M.tb*-infected cells decreased antigen export and increased the availability of antigen to the MHC class II antigen presentation processing, resulting in more effective CD4 T cell activation (Srivastava et al., 2016).

In addition, shuttling the mycobacterial antigens via EVs mediates the cross-presentation of mycobacterial antigens in distance. These EVs also contain MHC-I, MHC-II, and costimulatory molecules and it has been shown that they could active BCG-sensitized CD4+ and CD8+ T cells ***in vitro*** and naïve CD4+ and D8+ T cells ***in vivo*** (Giri and Schorey, 2008).

Moreover, when immature and mature DCs were exposed to these EVs, they induced the production of IFN-γ by autologous CD4+ T cells specific to *M.tb* antigens. The aforementioned results suggest that these EVs may act as antigen carriers and transfer mycobacterial proteins to the antigen-presenting cells (Vázquez-Flores et al., 2023).

In combination with these studies, the experiments by Smith et al. vividly confirmed the significant role of EVs as an extracellular source of antigen for T-cells during infection with *M.tb* (Smith et al., 2017). They utilized Rab27a-deficient mice as a model to investigate the role of EVs during TB infection. They observed that exosomes released during *M.tb* infection in mice significantly contributed to T-cell responses. Rab27a, a member of the Rab GTPases family (Elieh Ali Komi et al., 2020), is known to mediate exosome biogenesis and secretion (Ostrowski et al., 2010). The Rab27a-deficient mice exhibited reduced exosome concentrations in serum after TB infection, followed by an increase in bacterial burden. These changes were linked to the decreased mycobacterial antigen transport via exosomes, reduced activation of the lung and splenic T cell, and a decreased capacity to elicit a pro-inflammatory response. Additionally, the number of T cells producing IFN-γ upon antigen stimulation was also reduced (Smith et al., 2017).

### 3.6 Influencing the host immune cells’ metabolic pathways

During homeostasis, cells metabolize glucose mostly through the citric acid cycle (Krebs cycle) and oxidative phosphorylation (Lachmandas et al., 2016). However, during infection with *M.tb*, the bacteria perturb the host metabolism by inducing a metabolic switch toward aerobic glycolysis in CD14+ monocytes or CD3+ T cells isolated from human peripheral blood mononuclear cells (PBMCs). The metabolic shift was dependent on TLR2 and was facilitated by the activation of the AKT-mTOR (mammalian target of rapamycin) pathway (Lachmandas et al., 2016; Alipoor and Mirsaeidi, 2021). Regarding the presence and importance of EVs surface TLR ligands, it can be expected that these EVs could induce reprogramming of metabolic pathways in the recipient cells.

Our team previously reported that the exosomal miRNAs released by BCG-infected macrophages were enriched in a cluster of miRNAs that regulated host metabolism pathways (Alipoor et al., 2017). The cluster of dysregulated miRNAs upon infection with BCG included miR-1224, miR-1293, miR-425, miR−484, and miR−96 all were predicted to target metabolism and energy production-related pathways including the central carbon metabolism (CCM) pathways, genes involved in ketone body and amino acid synthesis, glycosaminoglycan biosynthesis and heparan sulfate/keratin sulfate metabolism pathways in recipient cells (Alipoor et al., 2017). Interestingly, this cluster of exosomal miRNAs also was enriched in the exosomes from the serum of TB patients (Alipoor et al., 2019a).

Modulation of metabolic exosomal miRNAs upon infection with mycobacteria suggests that mycobacteria may reprogram the expression of metabolic host miRNA network and metabolic-specific genes to recruit host metabolic pathways in favor of survival.

It has been shown that in TB patients, *M.tb* impacts the protein and lipid composition of circulating EVs, which vary according to the disease stage (Biadglegne et al., 2022). Therefore, *M.tb* may induce the enrichment of exosomes in a particular size and probably with a specific composition for modulating metabolic pathways for maintaining its survival (Singh et al., 2023).

### 3.7 The role of EVs on host-pathogen interactions and disease outcome

EVs from both the pathogen and the host could influence the outcome of the infection. It seems that *M.tb* takes advantage of these active bio-vesicles as the main strategy to control the host’s physiological condition and immune responses for its survival.

One strategy taken by *M.tb* is increasing the production of EVs by host cells (Walters et al., 2013; Alvarez-Jiménez et al., 2018; Singh et al., 2023) through manipulation of the host ESCRT-dependent and independent genes (Singh et al., 2023). Considering the role of TLRs in EV-mediated mechanisms, the enhanced secretion of EVs by infected cells may have a contrary effect. Exosomal TLR-2 ligands primarily trigger the activation of CD4+ T cells and the formation of granulomas, however, this is not sufficient to clear the bacterial infection because of the consequences of long-term TLR-2 stimulation mainly immunosuppression. This effect is exerted by promoting the secretion of immunosuppressive cytokines and finally suppression of MHC-II antigen presentation (Tapping and Tobias, 2003; Gehring et al., 2004; Pecora et al., 2006; Prados-Rosales et al., 2011).

On the other hand, *M.tb* inhibits T-cell receptor signaling by delivering lipoglycan to T-cells within mycobacterial EVs. Athman et al. observed that upon incubation of T cells with *M.tb* infected Macrophages, T cells were associated with mycobacterial LAM. This was also observed in the T-cells isolated from the lungs of tuberculosis-infected mice (mice were infected with *M.tb* via aerosol infection). In a series of experiments, they showed that the trafficking of bacterial LAM to T cells is via the bacterial EVs that are produced inside the phagosomes (Athman et al., 2017). They concluded that *M.tb* EVs escape from infected macrophages and enter the extracellular environment. These bacterial EVs can transport *M.tb* molecules to uninfected immune cells including T cells, emphasizing the significance of EV production during *M.tb* infection (Athman et al., 2015).

In addition, the concentration of mycobacterium EVs during infection depends on the presence of extracellular mycobacteria and varies in the different stages of infection in tuberculosis patients (Schorey et al., 2021). In the later stages of infection more extracellular rather than intracellular bacteria are expected to be found in the center of the granuloma which suggests that during these later stages of infection, Mycobacterium-derived EVs may have the higher concentration and so may exert relatively more biological effect (Schorey et al., 2021).

**Figure 2:**
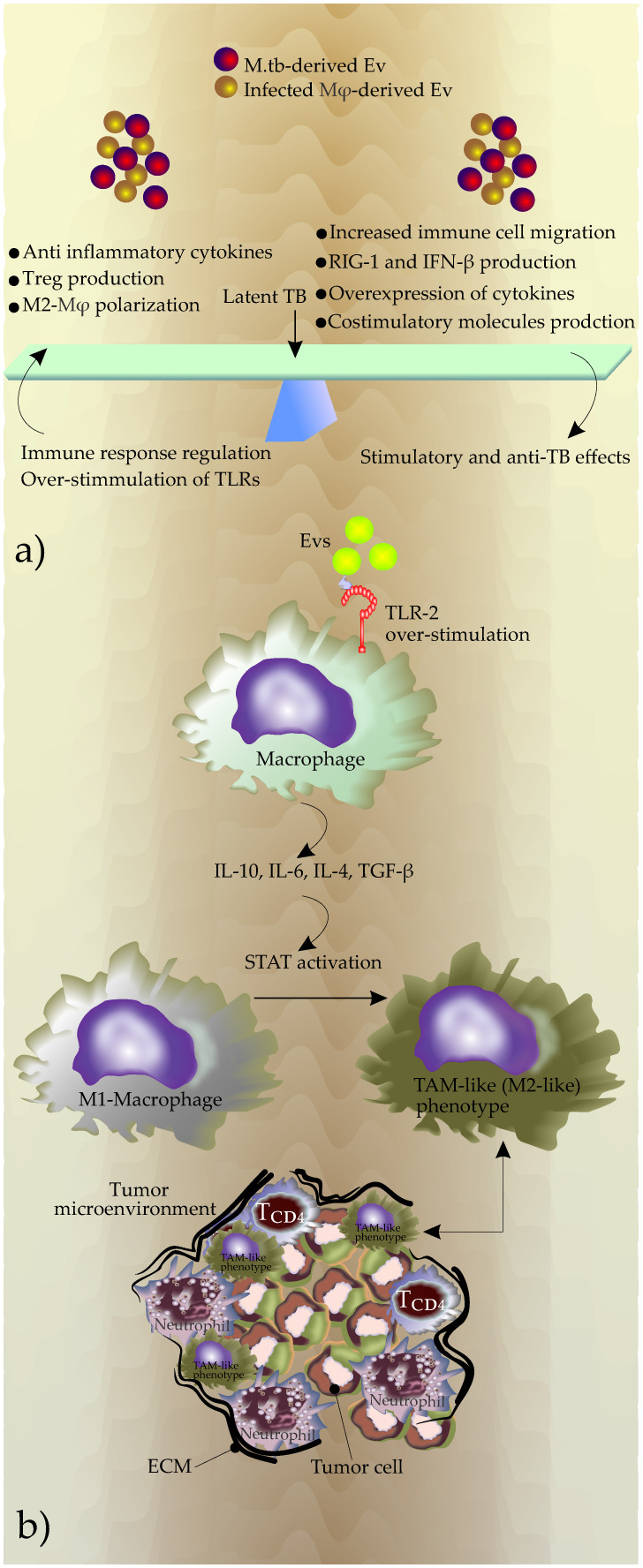
**(A)** The outcome of the EVs effects on the phenotype of diseases, **(B)** Potential mechanisms used by EVs to generate TAMs during chronic TB inflammation. Overstimulation of TLRs due to increased production of Evs induces expression of immune suppressive cytokines including IL-10, IL-6, IL-4, and TGF-β. This new cytokine profile promotes the polarization of macrophages in favor of a M2-like phenotype that in turn promotes the shaping of TME. The reciprocal interactions between TAM and the tumor microenvironment contribute to tumor progression.

It seems that bacteria strategically regulate the production, content, and release of these EVs to maintain the balance between immunity and immune evasion, which is a feature of latent *M.tb* infection and essential for the maintenance of bacterial intracellular survival (**Figure 2A**).

## 4 Potential roles of EVs in the promotion of tuberculosis towards lung cancer

Epidemiological research has shown that patients with TB have an increased risk of being diagnosed with pulmonary cancers with almost a two-fold increase in the risk of lung cancer (Shiels et al., 2011; Luczynski et al., 2022). Previous *in vitro* studies suggested that *M.tb* has the potential to promote cancerous processes and in the animal model, chronic tuberculosis infection could induce lung cancer in mice (Roy et al., 2021). Several mechanisms may be involved in this process including increased production of inflammatory cytokines which can target anti-apoptotic intracellular signaling pathways in the tumor cells. In addition, TB infection may cause a fibrotic change in the lung tissue, which increases the risk of lung cancer over time (Malik et al., 2022).

However, a remarkable feature of an inflammatory microenvironment is that it has the core required elements to induce the changes for cancer development (Alipoor et al., 2018; Komi and Redegeld, 2020; Liardo et al., 2021; Tian et al., 2022). In a chronic inflammatory site, the presence of several inflammatory cells and signaling molecules such as NF-KB, can increase the malignant progression of transformed cells due to their mutagenic predisposition. On the other hand, upon subverted host immune reactions during a persistent inflammation, the inflammatory cells and regulatory cytokines may attribute to increased angiogenesis and cell proliferation which promote tumor initiation and progression (Elieh Ali Komi and Jalili, 2022).

During a chronic TB infection, EVs may play the main role in changing the game in favor of a tumor microenvironment. During chronic inflammation, the content of EVs undergoes various changes, which may close them to the tumorigenic EVs (Liardo et al., 2021; Tian et al., 2022). These EVs shuttle a variety of different inflammatory molecules including noncoding RNAs or critical cancerous proteins in the inflammatory site that may change condition by different mechanisms including triggering tumorigenic pathways, epigenetic-related changes, macrophage polarization, TAM production, epithelial-mesenchymal transition (EMT), angiogenesis, etc (Alipoor et al., 2018).

### 4.1 Tumorigenic pathway

Noncoding RNAs are the most abundant and important regulatory molecules that are transferred via the EVs. These molecules have a paramount role in the immune phenotype of inflammatory niches (Alipoor et al., 2018). In silico analysis has shown that the dysregulated miRNAs in bovine tuberculosis have the potential to trigger the oncogenic pathways including mitogen-activated protein kinase, PI3K-Akt signaling, and fibroblast growth factors as the key factors (Ndzi et al., 2019a).

In another study, pathway analysis of the exosomal miRNAs released by the infected macrophages with BCG has shown that the target genes of these exosomal miRNAs are most associated with important cell cycle signaling pathways related to EGF/EGFR, WNT, Rap1, Ras, receptor tyrosine kinases ErbB, activated FGFRs, and VEGFA-VEGFR2 (Alipoor et al., 2017; Alipoor et al., 2019b). Disturbing these important pathways in cell proliferation and metabolism may make the recipient cells more susceptible to entering a cancerous stage. These clusters of *M.tb*-induced dysregulated miRNAs also were involved in amino acid, pyrimidine, and purine nucleotide biosynthesis which are increased in *M.tb*-infected lung tissue and are also an important factor in the progression of cancers (Alipoor et al., 2017). So, EVs released during *M.tb* infection have the potential to change the physiological condition in the recipient cells by targeting important pathways in cell cycle, metabolism, and proliferation that increase their susceptibility to tumor initiation and progression.

### 4.2 Epigenetic profile

TB exosomal miRNAs may also induce epigenetic changes inside the inflammatory niche which may create a suitable environment for tumor initiation.

miR-29a is one of the most common exosomal miRNAs in the serum of TB patients (Ndzi et al., 2019b). This miRNA belongs to the family of epi-miRNAs and regulates the methylation of DNA by targeting DNA methyltransferases (DNMTs) including DNMT1 or DNMT3 (Amodio et al., 2015). These events subsequently change the expression profile of the epigenetics enzymes including Enhancer of Zeste homolog 2 (EZH2).

EZH2, as the catalytic subunit of the Polycomb Repressive Complex 2 (PRC2), modifies gene expression by trimethylating Lys-27 on histone 3 (H3K27me3). This modification leads to the silencing of its target genes (Duan et al., 2020).

Activation of EZH2 increases the proliferation rate of lung cancer cells (Entezari et al., 2023), and promotes metastasis and EMT resulting in lung cancer progression. Elevated EZH2 levels and an increased count of genes silenced via H3K27me3 were linked to the advanced stage of lung cancer (Agarwal et al., 2016).

miR-21 is a known oncomir that plays a critical role in lung cancer, and it is reported that it is upregulated within the serum EVs from TB patients (Wang et al., 2011; Latorre et al., 2015; Duffy et al., 2018).

miR-21 promotes lung cancer via the regulation of different mechanisms involved in epigenetics modulation (Melnik et al., 2020), metastasis, genetic instability of tumor cells, angiogenesis (Ni et al., 2019), and EGFR-regulated anti-apoptotic factor (Seike et al., 2009). The mir-21 promoter is robustly regulated by STAT3, which is activated by IL-6 (Löffler et al., 2007). IL-6 is considered a major driver of disease severity in TB and a key player in the tumorigenesis mechanisms in Lung cancer. The IL-6/STAT3 pathway via miR-21 is an epigenetic switch linking inflammation to cancer (Iliopoulos et al., 2010) and may contribute to the initial stage of the tumor (Löffler et al., 2007; Bi and Chng, 2014; Liu et al., 2016; Saltarella et al., 2021). Increased cellular levels of miR-21 either by endogenous upregulation or uptake of miR-21-rich exosomes can trigger these tumorigenic mechanisms (Xu et al., 2020).

miR-31, miR-125b, miR146a, and miR-155 are also the most reported circulating miRNAs upregulated in the exosome obtained from TB patients (Daniel et al., 2022). These miRNAs are considered oncomiR since their target gene plays a role in the promotion of tumors (**Table 1**).

**Table 1:** The main cancer-associated miRNAs (OncomiRs) reported in EVs isolated from serum samples of TB patients.

Common Exosomal miRNAs in serum of TB patient	Biological role in promoting cancer
miR-31 (Kathirvel et al., 2020)	MicroRNA-31 promotes lung tumorigenesis, especially in mutant KRAS-driven lung cancer (Edmonds et al., 2016; Wang N. et al., 2017).
miR-125b	MicroRNA-125b functions as an oncogene in lung cancer cells (Amir et al., 2013; Wang X. et al., 2015; Wang N. et al., 2017)
miR146a (Chakrabarty et al., 2019)	miR-146a modulates different signaling pathways in lung cancer including TNF, NF-κB, MEK-1/2, and JNK-1/2 (Wani et al., 2021)
miR-155	Essential in regulating the immune system (Dezfuli et al., 2021) and in combination with miR-21 promotes non-small cell lung cancer (NSCLC) by downregulation of SOCS1, SOCS6, and PTEN (Xue et al., 2016)

Therefore, during a chronic TB infection, exosomal miRNAs derived from the infected cells may change the epigenetic and transcription profile inside the inflammatory niche and facilitate the development of the TME.

### 4.3 Macrophage polarization

During chronic TB, EVs may also change the phenotype of the inflammatory microenvironment by modulation of macrophage polarization. The increased production of EVs during TB infection and subsequently the continuous triggering of the TLRs by these EVs may start anti-immunity responses including an increase in the secretion of IL-10, IL-4, and TGF-β (Hirsch et al., 1996; Saraav et al., 2014). This event promotes polarization to TAM-like (M2-like) phenotype (Hu et al., 2016; Zhang et al., 2016) (**Figure 2B**).

TAMs play a significant role in tumor progression by producing substantial amounts of IL-10, TGF-β, and VEGF. These molecules facilitate immunosuppression and angiogenesis (Xu et al., 2020). Additionally, central transcription regulators, including STAT1, STAT3, and STAT6, which activate the M2 phenotype (Gong et al., 2017; Vergadi et al., 2017) may be modulated in the recipient cells by up-taking EVs during TB infection.

Triggering TLRs by EVs released by the infected macrophages may also increase the release of IL-6 (Flynn et al., 2019). IL-6 significantly increases invasion in lung cancer cells by regulating EMT (Liu et al., 2020). Additionally, IL-6 may stimulate the expression of IL-10, and together, macrophage polarization and TAM production (Dehai et al., 2014; Wang X. et al., 2017; Xu et al., 2020) (**Figure 2B**).

### 4.4 Epithelial-mesenchymal transition

It also has been shown that infection of human monocyte cells with *M.tb*, increases the invasion and induces EMT *in vitro* (Gupta et al., 2016). Additionally, TB EVs shuttle a significant amount of vimentin and HSPs (Diaz et al., 2016) which respectively regulate key events during EMT and impact malignant progression in lung cancer (Usman et al., 2021).

EVs derived from the Mtb-infected cells harbor an array of immuno-stimulatory and immuno-inhibitory factors that can contribute to cellular reprogramming in recipient cells. These EVs play a pivotal role in regulating intracellular communication within the inflammatory niche during chronic tuberculosis. Additionally, they can modulate their surroundings, creating an optimal microenvironment for tumor initiation and progression (Schorey and Bhatnagar, 2008).

## 5 Clinical potential: therapeutic and diagnostic role of EVs in mycobacterial infection

The World Health Organization (WHO) established the strategy against TB with the ultimate objective of TB by 2035. However, diagnosis of tuberculosis is still challenging, and we need to accelerate case finding and early TB diagnosis for which finding suitable biomarkers is crucial. Exosomal biomarkers are ideal because their surrounding membranes provide stability to their sensitive content. Furthermore, they are easily separated from nearly all biofluids, allowing for less expensive investigations, and avoiding the need for extensive biopsy collection.

Exosomal biomarkers have been intensively investigated in the context of tuberculosis. Many studies explored and compared the cargo from the exosome obtained from cell culture media, serum of TB patient as well as animal models to find a suitable biomarker for diagnosis of TB. These components encompass signatures of lipids, proteins, various metabolites, and miRNAs. They have been identified through unbiased “omics” discovery methods and extensively investigated by several studies (Kruh-Garcia et al., 2015; MacLean et al., 2019; Mirzaei et al., 2021; Wykowski et al., 2021; Nan et al., 2023).

Despite the promising results demonstrated by exosomal biomarkers, research findings remain inconsistent, and significant limitations persist in translating these biomarker discoveries into clinical applications.

EVs also attracted attention owing to their potential to be used as an *M. tuberculosis* vaccine. EVs have both adjuvant and antigenic properties and stimulate a strong immune response in the recipient macrophages (Giri and Schorey, 2008). The intranasal vaccination using EVs released from *M.tb*-infected macrophages, increased the generation of memory T cells in mice (Cheng and Schorey, 2013). Likewise, the EVs collected from the cell culture media of the macrophages infected with *M.tb* culture filtrate proteins (CFP) showed a good potential as TB vaccine compared to BCG. These EVs evoked T cell response *in vitro* and *ex vivo* (Giri et al., 2010) and also in mice models showed a comparable decrease in bacterial survival compared to BCG vaccinated mice. Likewise, mice boosted with CFP-related EVs and BCG exhibited a notable reduction in bacterial counts within the lung upon challenge with TB (Cheng and Schorey, 2020).

The EVs in tuberculosis infection also have shown promising potential in therapy. Interestingly in a recent study, the EVs released by *M.tb*-infected macrophages in combination with moxifloxacin, synergistically increased *M.tb* clearance in macrophages *in vitro* and *in vivo* (Cheng et al., 2021). Moxifloxacin is a key antibiotic against MDR-TB. This finding highlights the importance of EV-based therapy methods as a new strategy in the battle against tuberculosis.

It has shown that upon vaccination, BCG directly altered the transcriptome of hematopoietic stem cells (HSCs) in bone marrow towards the increased expression of myelopoiesis-related genes by epigenetic reprogramming of these cells (Kaufmann et al 2018).

These epigenetic alterations increased the transcription of the cytokines genes such as IFN-γ, TNF, and IL-1b which are essential for protective antimycobacterial immunity. This process subsequently enhanced the production of epigenetically modified macrophages with significantly better protection ability against virulent *M.tb* infection. It is suggested that long non-coding RNAs (lncRNA) play the main role in this context by influencing the mechanisms of epigenetic reprogramming (Kaufmann et al., 2018; Cirovic et al., 2020). Since lncRNAs are packaged inside the EVs in high amounts, it can be hypothesized that EVs released by mycobacterium-infected cells may transfer these specific lncRNA to the naïve monocytes and mimic these effects in these cells which is expected to increase the ability of the recipient naïve monocytes to better clearance of the bacteria after maturation to macrophages. Further studies are needed for a better understanding of the mechanisms of EVs in this context.

## 6 Conclusion and unmet questions

The paramount role of EVs in the pathogenesis of bacterial infection and host immunity has been highlighted. It is well known that during infection with *M.tb*, both bacteria and infected host produced EVs containing bacterial components. These EVs have several (and different) mechanisms to interact with the immune system including binding to TLRs or stimulating RNA sensors in host cells.

One important open question in this context is how different populations of EVs derived from the bacteria or infected host are linked to the phenotype of disease and what is their role in host immunity against the pathogen. We also need to define the heterogeneity of immune responses to the different populations of EVs. We know that the content of EVs changes over time during infection and in different stages of infectivity, but we don’t know yet how the process of exosomal packaging during infection is controlled by bacteria or host.

Understanding the specific contents of these EV populations as well as their specific surface receptors could help to address these questions. To achieve this, we first require sophisticated methods for efficiently separating various EV populations. Creating infection models is another tactic to investigate how intracellular infections produce EVs in the host environment. Selectively inhibiting EV release from the bacteria or host cells could be an effective tactic to evaluate the outcome of infection related to each EV population. Although our knowledge of EVs’ role in the pathogenicity of bacteria is still limited, gaining more expertise in this area can help us to identify potential novel therapeutic approaches based on EVs.

## Author contributions

SD: Supervision, Writing – original draft, Writing – review & editing. DE: Visualization, Writing – review & editing.
